# Microbiological Changes in Meat and Minced Meat from Beavers (*Castor fiber* L.) during Refrigerated and Frozen Storage

**DOI:** 10.3390/foods10061270

**Published:** 2021-06-02

**Authors:** Monika Ziomek, Łukasz Drozd, Michał Gondek, Renata Pyz-Łukasik, Francesca Pedonese, Mariusz Florek, Piotr Domaradzki, Piotr Skałecki

**Affiliations:** 1 Department of Food Hygiene of Animal Origin, University of Life Sciences in Lublin, Akademicka 12, 20-950 Lublin, Poland; monika.ziomek@up.lublin.pl (M.Z.); michal.gondek@up.lublin.pl (M.G.); renata.pyz@up.lublin.pl (R.P.-Ł.); 2 Department of Veterinary Sciences, University of Pisa, Viale delle Piagge 2, 56124 Pisa, Italy; francesca.pedonese@unipi.it; 3 Faculty of Animal Sciences and Bioeconomy, Institute of Quality Assessment and Processing of Animal Products, University of Life Sciences in Lublin, Akademicka 13, 20-950 Lublin, Poland; mariusz.florek@up.lublin.pl (M.F.); piotr.domaradzki@up.lublin.pl (P.D.); piotr.skalecki@up.lublin.pl (P.S.)

**Keywords:** minced meat, game meat, microbiological profile, *Castor fiber* L.

## Abstract

This study aims to evaluate the microbiological status, pH, and water activity of European beaver meat to establish its shelf-life and microbiological safety. In this study, the microbiological profiles of meat and minced meat obtained from the carcasses of beavers were investigated. Microbial evaluation of the chilled meat was performed within 24 h after hunting, on the 7th day and 14th day, and the evaluation of the frozen meat was made during the 11th week of storage. Meat samples were analysed for total viable count (TVC), psychrotrophic bacteria count (PBC), *Enterobacteriaceae* count (EBC), *Escherichia* *coli* count (EC), total staphylococcal count (TSC), lactic acid bacteria count (LABC) and total yeast and mould counts (TYMC). Tests for the presence of pathogenic bacteria from the genus *Salmonella* and *Listeria* were also performed. Additionally, the pH and water activity were determined. The initial amount of TVC was 4.94 log CFU/g in meat samples and 4.80 log CFU/g in minced meat. After 14 days of storage, the TVC increased to 8.33 in meat samples and 8.08 log CFU/g in minced meat. Pathogenic bacteria such as *Listeria* and *Salmonella* were not found in the beaver meat tested. The microbiological state of meat stored frozen for 11 weeks was comparable to the state found in meat stored refrigerated for seven days regarding the number of microorganisms.

## 1. Introduction

The beaver (*Castor fiber* L.) is the largest living rodent in Europe and the second largest in the world after the capybara. It is an aquatic and terrestrial animal, has a nocturnal lifestyle and shows strong territoriality. In Poland, according to the regulation of the Polish Ministry on Environment from December 2016, the beaver population is under partial species protection [1]. The inclusion of beavers on the list of endangered species and the “Active Beaver Protection Programme” led by the Research Station of the Polish Academy of Science in Popielno and the Polish Hunting Association led to significant growth in the beaver population in Poland. Over the years, from 2000 to 2018, a 4-fold increase in the population’s size was observed. According to data from Statistics Poland [2], in 2018, the population of beavers in Poland consisted of about 125,000 individuals. For this reason, the Minister for the Environment agreed to a partial reduction. This reduction is by culling, which is permitted through shooting with hunting weapons or trapping in a strictly defined season from 1 October to 15 March. The number of beaver individuals to be shot in Baltic Sea region countries is increasing from year to year [3]. As a result, beaver meat may become more available on the food market. Beaver meat is classified as game and is not a popular food species. However, this meat has well-appreciated culinary properties and was once considered a delicacy, especially the beaver’s hind legs, liver and tail fin, to which aphrodisiac properties were attributed. Beaver meat can become a constituent of processed meat products, i.e., sausages, burgers, patties or meatballs made of different types of meat. Therefore, it seems appropriate to define its quality. In general, the meat quality concept includes eating pleasure, good appearance, physical properties, quality, nutritional value and safety. The physicochemical properties and nutritional value of beaver meat have already been described [4,5,6,7]. Beaver meat contains 21.50 ± 1.01% of protein. The average percentage of indispensable amino acids reaches 45.00%, and at the highest content in mussels are lysine (1.85 g/100 g) and leucine (1.65/100 g) and within nonessential amino acids glutamine acid (3.49 g/100 g) and aspartic acid (1.89 g/100 g). The percentage of intramuscular fat from skeletal muscle is 1.99 ± 1.13%. The predominant class of fatty acids is polyunsaturated fatty acids (averaging 49.39%), followed by saturated fatty acids (27.81%) and monounsaturated cis fatty acids (15.96%). Linoleic acid (LA, 18:2 n-6) is the most abundant fatty acid in intramuscular fat (>34% of total). The pH value of beaver meat changes during cold storage and ranged from 5.61 after 24 h to 6.10 after 7 days [8,9]. Therefore, there would seem to be merit in undertaking research assessing the safety of this meat and its resistance to deterioration when stored in refrigerated conditions. There is little information available in the literature on biological hazards related to the consumption of beaver meat. The most widely described disease borne by it is Tularemia caused by *Francisella tularensis*. Tularemia may be derived from inadequately cooked beaver meat [10]. Beavers are also indicated as a source of the *Echinococcus multilocularis* and *Echinococcus granulosus* tapeworms, and *Trichinella* spp. roundworms [11,12,13,14,15]. The microbiological quality of game varies greatly and depends on many factors, e.g., the microflora on the surface and in the digestive tract of animals; the hunting methods; the location of the projectile injury; the place where the game was hunted; the time needed, a technique used, and hygienic conditions provided during gutting; the temperature, duration and hygiene during transport to the cooling point; and technical and sanitary conditions during skinning, butchering of hunted prey carcasses [16,17,18,19]. The risk of microbiological contamination of game meat after hunting is reduced if the animal is quickly found and eviscerated [16]. Studies show that immediately after hunting, the number of microbes in the meat is negligible. It should, therefore, be assumed that the microbiological status of wild meat will vary widely.

It should also be stressed that other factors, such as the chemical composition of meat, gaseous environment and nutrient availability, as well competition from other bacteria, also affect how any particular bacteria may grow in this matrix [20].

Minced meat is produced from fresh or frozen skeletal muscles together with the fatty tissue attached to them. In the mincing process, the cellular structure of the muscle tissue is irretrievably lost, and as a result, minced meat becomes a highly nutritious and perishable product. Due to redistribution of surface contamination throughout the whole mass, such a product is in jeopardy of rapid bacterial growth. For the meat industry, the question of how long the maximum storage time of raw meat can be from slaughter until mincing is still unresolved [21].

Taking into account the growing population of beavers in Poland and the possibility of hunting these animals, it is expedient to determine the microbiological status of beaver meat in the context of microbiological criteria established by food law and commonly used parameters for microbiological quality assessment of game. Therefore, the objective of the pilot study is to evaluate common food-borne and food spoilage bacteria prevalence in beaver meat and its shelf-life, as indicated by the change in microbiological contamination, water activity and pH value during storage.

## 2. Materials and Methods 

### 2.1. Sampling

The research material was prepared from male European beavers (*n* = 6). The animals were shot under reduction hunting by authorised hunters as members of the Polish Hunting Association from 5 to 14 March 2017 in controlled hunting zones inside Lublin voivodeship under two individual grants of permission (WPN.6401.33.2016.KM of 7 Apr 2016 and WPN.6401.85.2016.KM of 4 July 2016) and a regulation made by the Regional Director for Environmental Protection in Lublin on 30 November 2016 (the Official Journal of Lublin voivodeship of 1 Dec 2016, item 4828). After hunting, beavers were bled, eviscerated, skinned and weighed. An appropriately trained person examined the body and viscera of animals according to Chapter III of Section IV of Annex III to Regulation [22]. During the examination, no visible anomalies or signs suggestive of the meat posing a health risk were noticed. Eviscerated carcasses were transported to the laboratory in insulated containers within 24 h of shooting in compliance with the highest hygienic standards and keeping temperature throughout the meat below 4 °C. The head, paws and tail fin were removed from each animal. For the purposes of this study, the left and right thighs were detached from each carcass, then deboned and trimmed of visible adipose and connective tissue. The principal muscles were collected from individual hind legs: the *semimembranosus*, *semitendinosus*, *biceps femoris*, *gluteus medius*, and *quadriceps femoris*. The muscles from the left thigh of each beaver were divided randomly into four separate samples and wrapped individually using sterile re-sealable polyethene bags (Fisherbrand™, 540 mL). The muscles from the right thighs were minced in sanitary conditions using a Predom-Mesko device (Poland) and divided into four individual samples. The samples thus prepared were stored refrigerated at 4 °C or frozen at (−18 °C) until they were analysed. Physicochemical measurements and microbial evaluation of refrigerated whole-muscle tissue and minced muscle tissue were performed initially within 24 h after hunting and subsequently on the 7th and 14th days. For frozen tissue, the evaluation was performed at the end of the 11th week of storage (−18 °C). Before analysis, frozen meat samples were thawed for 24 h at a temperature of 4 °C. In total, 48 beaver meat samples were tested, 24 each of muscles and minced meat (*n* = 48).

### 2.2. Microbiological Analysis

For all microbiological counts, 10 g samples from each re-sealable bag were aseptically weighed into sterile lab bags (BagFilter 400 mL, Interscience, Saint Nom la Brétèche, France) with 90 mL of dilution fluid–saline peptone water (BioMaxima, Lublin, Poland) and then were homogenised in a stomacher (Seward, Worthing, UK) for 2 min at the normal speed of 230 rpm and as required by the PN-EN ISO 6887-3:2005 and PN-EN ISO 7218:2008 standards [23,24]. Other decimal dilutions were prepared from the 10^−1^ dilution and were plated onto appropriate media. The samples to provide TVC and PBC were cultivated on Plate Count Agar (BioMaxima, Poland) and enumerated after incubation at 30 °C for 48 h and 0–4 °C for 14 days, respectively [25,26]. Bacteria to be the source of EBC data were enumerated in Violet Red Bile with Lactose Agar (BioMaxima, Poland) after incubation at 37 °C for 24 h [27]. The EC was determined using Tryptone Bile X-glucuronide Agar (BioMaxima, Poland) as the medium. Plates were incubated at 44 °C for 24 h [28]. The LABC was facilitated by bacterial culture using de Man, Rogosa and Sharpe (MRS) agar (BioMaxima) as the medium. Plates were incubated at 30 °C for 48 h [29]. The TSC was estimated after culture using Baird-Parker agar and Brain Heart Infusion (Biomaxima, Poland) broth (37 °C for 24 h in both cases) [30]. For the confirmation of coagulase-positive staphylococci, Rabbit Coagulase Plasma (Pro-Lab Diagnostics, Wirral, UK) and the SENSILAtest STAPHY 24 identification kit (Erba Lachema, Brno, Czech Republic) were used. The TYMC was ascertained through culture on an agar medium with Dichlorate Rose Bengal and Chloramphenicol LAB-AGAR (BioMaxima, Poland) [31]. The culture was incubated at 25 °C for five days. Isolation and determination of *Salmonella* spp. were performed according to PN-ISO 6579:2003 [32]. Twenty-five grams of a sample in 225 mL of buffered peptone water was homogenised in the stomacher and incubated at 37 °C for 18 h. After that, Rappaport–Vassiliadis broth (incubated at 41.5 °C for 24 h) and then xylose lysine deoxycholate (XLD) and brilliant green agar (BGA) (incubated at 37 °C for 24 h) were used. If no typical colonies appeared on XLD and BGA media, the test was discontinued according to the stipulations for the procedure in the ISO standard. In order to determine the presence of *Listeria* spp., an examination was performed guided by PN-EN ISO 11290-1 [33]. Twenty-five grams of a sample in 225 mL of half Fraser broth was homogenised in the stomacher and incubated at 30 °C for 24 h. Then, the homogenate was added to the Fraser broth, and the solution was incubated at 37 °C for 24 h. Subsequently, incubation took place again in Ottaviani and Agosti medium (ALOA) and Oxford medium at 37 °C for 24–48 h. Confirmatory tests were performed (Gram staining, mobility capacity, sugar fermentation, and hemolysis tests). Analyses were carried out on the materials from each re-sealable bag separately. All bacterial populations were determined as the log of colony-forming units (log CFU g^−1^).

### 2.3. pH Measurement 

The pH was measured in triplicate using a CP-401 portable pH-meter (Elmetron, Poland) and an ERH-12-6 penetrating glass electrode (Hydromet, Poland) calibrated at the 2 points pH 4.00 and pH 7.00 with high-accuracy pH buffer solutions (±0.02 at 20 °C; Elmetron, Poland). The pH meter allowed for the automatic detection of buffer solutions and automatic temperature compensation.

### 2.4. Water Activity Measurement

The water activity was measured in triplicate using a HygroLab C1 water activity meter (Rotronic, Bassersdorf, Switzerland). Measurements were made using the AWQ mode and allowing stabilisation for 15 min after samples had reached room temperature.

### 2.5. Statistical Analysis

A statistical analysis of the data was performed using Statistica 13 (Tibco, Palo Alto, CA, USA). Statistical differences were determined by one-way analysis of variance (ANOVA) and Tukey’s post-hoc test. A *p*-value of ≤0.05 was considered statistically significant for all comparisons. All data are presented as means ± SD.

## 3. Results

### 3.1. Bacterial Contamination of Carcasses 

In the studies carried out, a statistically significant increase in the number of microbes was found from earlier to later days of cold storage in all the bacteria studied: TVC, PBC, EBC, EC, LABC, TSC, and TYMC (Table 1). The TVC in the meat samples tested showed an upward trend from 4.94 to 8.33 log CFU/g. In parallel with the lengthening time of storage of meat, the PBC increased from 3.98 to 8.46 log CFU/g. The EBC rose from 3.27 to 5.70 log CFU/g and the EC from 3.11 to 4.80 log CFU/g. LABC were detected in the meat samples in the number of 3.68 to 6.01 log CFU/g. The TSC increased from 3.03 to 4.46 log CFU/g and TYMC from 3.11 to 4.80 log CFU/g. There was a statistically significant rise in the number of bacteria except for TVC in the samples of frozen meat in all bacterial counts taken, and the PBC was comparable to that determined on the seventh day of cold storage.

In Table 2, the numbers of microorganisms (TVC, PCB, EBC, EC, LABC, TSC, and TYMC) are shown, which were detected in minced beaver meat on 24 h, 7 and 14 days of refrigerated storage and in frozen minced meat after 11 weeks of storage. The growth of microorganisms in the minced meat during storage showed similar trends to those in the stored samples, which had not been minced. The TVC in refrigerated minced meat rose from 4.80 to 8.08 log CFU/g. The PBC also showed proliferation from 3.62 to 8.33 log CFU/g. Significant increases were also observed in other microbe counts. The EBC increased from 3.34 to 5.70 log CFU/g and the EC from 1.34 to 1.70 log CFU/g. The growth in LABC was from 3.4 to 5.66 log CFU/g, that of the TSC from 3.00 to 4.27 CFU/g and the rise in the TYMC was from 3.22 to 4.80 CFU/g. In frozen minced meat, bacterial growth in all groups apart from PBC was comparable to that observed on Day 7 of refrigeration. The studies carried out did not reveal statistically significant differences in the number of relevant microorganisms on individual storage days between minced meat and meat with preserved tissue structure.

### 3.2. pH and Water Activity Changes

**Figure 1:**
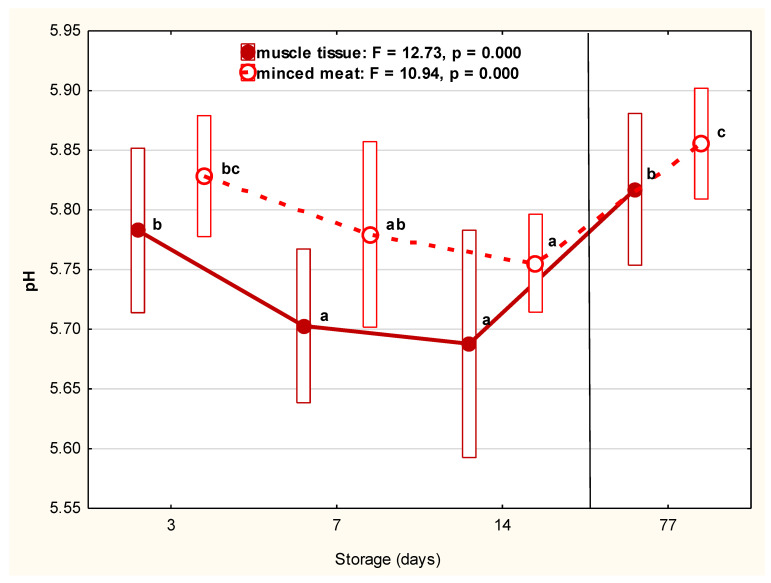
The pH of muscle tissue and minced meat of beaver during refrigerated (1, 7, 14 days) and frozen (77 days) storage. Red filled dots represent the average pH value of muscle tissue. The continuous line represents the trends in changes between storage days. The red empty dots represent the average pH value in minced meat. The dotted line represents the trends in changes between storage days. Bars represent the standard deviation. The mean values marked with letters (a, b, c) differ statistically at *p* ≤ 0.05.

A significant (*p* < 0.000) decrease in pH was observed during refrigerated storage up to Day 14 (to 5.69 for whole-muscle and 5.75 for minced tissue) (Figure 1). However, the highest pH values were determined after 11 weeks of frozen storage (5.82 for muscle tissue and 5.86 for minced meat). Nevertheless, the pH measurements obtained in the present research indicate the correct level of meat acidification from the technological and hygienic point of view.

**Figure 2:**
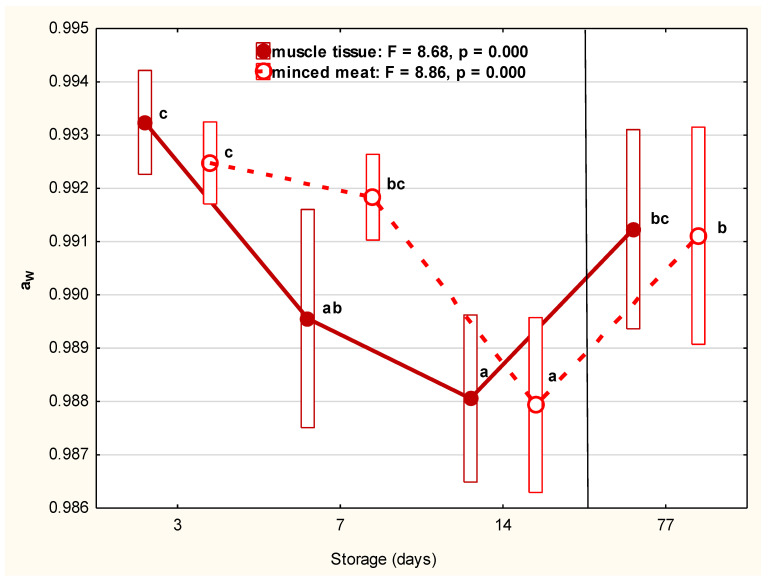
The water activity of muscle tissue and minced meat of beaver during storage. The a_w_ of muscle tissue and minced meat of beaver during refrigerated (1, 7, 14 days) and frozen (77 days) storage. Red filled dots represent the average a_w_ value of muscle tissue. The continuous line represents the trend in changes between storage days. The red empty dots represent the average a_w_ value in minced meat. The dotted line represents the trend in changes between storage days. Bars represent the standard deviation. The mean values marked with letters (a, b, c) differ statistically at *p* ≤ 0.05.

Refrigerated storage duration up to Day 14 significantly (*p* < 0.000) decreased water activity (a_W_) in both groups of examined beaver meat, from 0.993 to 0.988 for whole-muscle tissue and from 0.992 to 0.988 for minced meat. In turn, the level of a_W_ after frozen storage over 11 weeks (0.991) did not differ significantly from those on 24 h after shooting (0.993) and 7 (0.990) for refrigerated samples of muscle tissue and was similar to that on Day 7 (0.992) for minced meat (Figure 2).

## 4. Discussion

Many factors associated with post-mortem treatment of the carcasses determine the microbiological parameters of game meat and as a consequence play an important role in its initial hygienic status. The initial microbiological status of the meat has a significant impact on how safe it is and how long the storage period can be [16,17,18,34]. It is well known that the initial microbiological status of the meat determines the safety of meat for the consumers as well as length of its storage period. The TVC, EC and presence of *Salmonella* were evaluated to establish compliance with the European food law requirements [35]. Additionally, commonly used food hygiene criteria such as EBC, PBC, LABC, TYMC and presence of *Listeria* were also evaluated. The EBC is used for determining the level of microbiological contamination of carcasses and meat derived from them, while the total numbers of PBC and LABC determine the hygienic status of refrigerated meat. In contrast, the TYMC and presence of *Listeria* found in the carcasses and meat indicate that the source of these microorganisms should be linked with the environment in which post-slaughter dressing and dismemberment of carcasses were performed.

### 4.1. The Microbiological Parameters

There are currently few data available on the contamination of beaver meat. The TVC in the meat of beavers obtained 24 h after their shooting in Lithuania was found to be 3.94 log CFU/g [9]. In the studies performed for this investigation, the TVC and PBC in beaver meat on 24 h after shooting were comparable to those yielded in a muscle (*M. longissimus dorsi*) of male deer in north-eastern Poland, where the TVC was 5.10 log CFU/g and the PBC 3.80 log CFU/g, which may indicate a comparable level of overall microbiological contamination of game meat [36]. The TVC exhibited a rising trend during refrigerated storage. Similar relationships were found during the storage of meat obtained from the hind leg and shoulder of wild boars [37].

*Enterobacteriaceae*, including *E. coli*, are a typical indicator of faecal contamination, and their presence in muscle tissue may primarily indicate the presence of intestinal pathogens and accelerate the process of meat spoilage. In the case of game animals, the presence of *Enterobacteriacea* bacteria may also indicate that the animal was killed with poor shot placement, as well as indicate delayed gutting [38]. It has been confirmed that contamination with *Enterobacteriaceae* in the carcasses of wild animals shot unskilfully is significantly higher than contamination in carcasses of animals killed with good shot placement [16,39]. The average *Enterobacteriaceae* contamination found in the meat of beavers shot in Lithuania was 1.84 ± 0.11 log CFU/g at 24 h post kill [9]. In studies of wild boar meat shot in Poland, lower numbers of *Enterobacteriaceae* were found: 1.69 log CFU/g after 48 h and 2.43 log CFU/g after 360 h of storage at 2 °C [40]. Contamination with *Enterobacteriaceae* in wild animal meat is highly variable. This is confirmed by studies of fresh wild boar meat from southern Italy, where the level of *Enterobacteriaceae* in individual individuals ranged from 0.00 to 5.67 log CFU/g [39]. During the storage of fresh meat of wild animals, microorganisms of the *Enterobacteriaceae* family may constitute an important part of the overall microbial contamination. The ratio of the number of *Enterobacteriaceae* to the total number of microorganisms in studies carried out in Latvia was constant across all storage periods of roe and red deer meat and accounted for 60% of the total number of bacteria [41].

Lactic acid bacteria are widespread in the natural environment as well as in the production environment, and their presence on carcasses and meat is unavoidable. Heterofermentative strains of *Lactobacillus* (*Lactobacillus* spp., mainly *L. curvatus* and *L. sakei*), heterofermentative *Leuconostoc* (*Leuconostoc* spp.) and *Carnobacterium* spp. are the principal microorganisms that cause meat spoilage. The multiplication of heterofermentative strains of LAB during meat storage causes organoleptic changes, i.e., in smell and colour, and leads to the formation of threads of slime (ropy slime formation) [42]. The reason for these changes is in the production of a significant number of undesirable catabolites, such as CO_2_, ethanol, acetic acid, butanoic acid, and acetoin. The available literature notes a level of contamination of beaver meat with LAB bacteria of 1.38 log CFU/g 24 h after the shooting [9]. In wild boar meat, the amount of lactic acid bacteria in fresh samples ranged from 0.00 to 5.61 log CFU/g, which indicates a wide variation in the contamination of wild boar with lactic acid bacteria [39].

One of the causes of human food poisoning is the presence in food of staphylococcal enterotoxins produced by coagulase-positive *S. aureus* strains. The presence of coagulase-positive *Staphylococcus aureus* spp. *aureus* was confirmed in only one sample of minced meat. In the studies presented by Lawson et al., species analysis was performed on catalase-negative *Staphylococcus* strains isolated from European beavers, and it confirmed the new *Staphylococcus* species *Streptococcus castoreus* sp. *nov.* [43].

Game meat can also be a source of pathogenic *Salmonella* spp. and *Listeria* spp. bacteria. Studies of fresh wild boar meat from southern Italy have shown the presence of *Salmonella* bacilli in almost one-third of samples (31.82%) [39]. The results of the studies presented by Atanassova show that game meat can be contaminated with *Listeria* spp. bacteria. *Listeria* was confirmed in 4.8% of the game meat samples examined [16]. The studies carried out did not reveal the presence of microorganisms of the *Listeria* or *Salmonella* genus. Similar results were obtained in studies of wild boar meat, which also did not detect the presence of these bacteria [40].

Current EU food law imposing microbiological criteria does not set down hygiene requirements for wild animal carcasses. However, the regulation does adopt as law a food safety criterion for minced meat and raw meat products produced from the meat of species other than poultry and intended for consumption after heat treatment. This criterion is the absence of *Salmonella* in 25 g. According to this criterion, each test batch of minced meat should be considered satisfactory if no *Salmonella* bacilli are found in any of the five samples of 25 g (representing the batch). The hygienic criteria that signal the level of microbiological contamination of minced meat are TVC and EC. The established contamination limits defined by the limit (m) and maximum (M) number of microorganisms per gram of minced meat are as follows: m = 5 × 10^5^ CFU/g (5.7 log CFU/g), M = 5 × 10^6^ CFU/g (6.7 log CFU/g) for TVC; and m = 50 CFU/g, M = 500 CFU/g for *E. coli*. The hygienic quality of the process is considered unsatisfactory if at least one of the five contamination values indicated for the presence of the listed microorganisms exceeds M or if more than two of the five contaminant values indicated fall between m and M [35]. 

In the studies carried out, it was found that freezing the beaver meat slowed down the growth of the microorganisms studied. The microbiological state of meat stored frozen for 11 weeks was comparable to the state found in meat stored refrigerated for seven days regarding the number of microorganisms. However, our research showed an increase in the number of microorganisms in all bacteria studied in the frozen meat compared to the number of microorganisms found 24 h after shooting. The multiplication of microorganisms can explain the microbial growth in frozen meat during the thawing process. According to Leygonie et al., during thawing, microorganisms intensively grow due to very favourable growth conditions, especially thanks to the presence of readily available nutrients from thawed meat. In addition, since thawing is a slower process than freezing, some parts of meat may be exposed to more favourable conditions for microbial growth than others [44]. The moisture lost during thawing is rich in proteins, vitamins and minerals derived from the structural disarray caused by the freezing process, which consequently provides an excellent medium for microbial growth [45].

The findings of this study have to take into consideration some limitations. Firstly, there are no current EU food law governing requirements for meat cuts. Therefore, in order to assess the microbiological status of beaver meat cuts, we had to use other available literature sources, such as studies concerning various species of game and food-producing animals. Carcasses of game animals, including beavers, are available only at a certain time of each year and in a certain, small amount. The material collected for research, therefore, has a limited sampling scale.

### 4.2. pH and Water Activity

The results of pH values obtained in the present research are higher than those reported by Klupsaite et al. for beaver *Gluteus medius* 24 h post-mortem (5.61) but lower than values reported previously by Florek et al. for *Semimembranosus* after 7 days’ storage (6.10) [5,9]. Allen et. al., reported in poultry breast very low or not significant correlations between pH and bacterial counts [46,47]. Similarly, Byun et al., found only medium correlations between pH and bacterial counts during the storage of fresh pork and beef [48]. In turn, in accordance with the opinion of Bruckner et al., meat pH as an intrinsic factor can be disregarded as an influence on the shelf life of fresh pork and poultry [49].

To the best of our knowledge, no study has previously determined the water activity in beaver meat. Reid et al., observed a decrease in a_W_ from 0.96 to 0.93 in vacuum-packed beef primal cuts during chilled commercial storage for two weeks. In turn, values of a_W_ noted in the present study were comparable to the range (between 0.990 and 0.992) reported by Bruckner et al., for fresh poultry and pork stored at 4 °C for approximately 14 days. However, no significant influence of the a_W_ on the shelf life of pork or poultry was found [49,50].

## 5. Conclusions

The studies proved that the microbiological quality of beaver meat with respect to all the bacteria studied was satisfactory 24 h after shooting as well as up to the 7th day of refrigeration storage and up to 11 weeks of freezing. During the above-mentioned time periods, the minced beaver meat met the hygienic (TVC and EC) and food safety criteria (absence of *Salmonella*) established in Regulation 2073/2006, and it should be processed and consumed.

The high counts of microorganisms found 24 h after shooting may be related to the prevailing hygienic conditions during the evisceration, skinning and preparation of the carcases of hunted beavers. During the proceedings of the hunted beavers, hunters should make efforts to provide the best hygienic conditions during the handling of beavers’ carcases as soon as possible after the animal has been shot.

Pathogenic bacteria such as *Listeria* and *Salmonella* were not found in the beaver meat tested. Due to the non-detection of these bacteria, beaver meat can be assessed as safe in terms of the presence of these pathogens.

Based on a significant number of staphylococci in beaver meat, it can be concluded that it will be worth carrying out a thorough species analysis of bacteria of the *Staphylococcus* genus in beaver meat in the future. 

## Figures and Tables

**Table 1:** Microbiological counts (log CFU/g and % TCV) of a different microbial group of beaver meat samples (*n* = 6) *^a^*.

Microorganism *^b^*	Time of Storage			Weeks of Frozen
	24 h	7 Days	14 Days	11
TVC	4.94 ± 0.17 A	7.07 ± 0.45 B	8.33 ± 0.25 C	5.97 ± 0.27 D
PBC	3.98 ± 0.44 A	5.54 ± 0.31 B	8.46 ± 0.24 C	4.71 ± 0.28 D
EBC	3.27 ± 0.29 A	4.91 ± 0.13 B	5.70 ± 0.19 C	3.82 ± 0.38 B
EC	1.37 ± 0.27 A	1.54 ± 0.18 AB	1.72 ± 0.15 B	1.52 ± 0.25 AB
LABC	3.68 ± 0.37 A	5.17 ± 0.47 B	6.01 ± 0.52 C	4.51 ± 0.19 B
TSC	3.03 ± 0.13 A	3.74 ± 0.16 B	4.46 ± 0.32 C	3.68 ± 0.12 B
TYMC	3.11 ± 0.29 A	4.29 ± 0.53 B	4.80 ± 0.15 C	3.77 ± 0.12 B

*^a^* Within rows, means with different letters are significantly different (*p* < 0.05). *^b^* TVC, Total Viable Count; PBC, Psychrotrophic Bacteria Count; EBC, *Enterobacteriaceae* Count; EC, *E. coli* count; TSC, Total Staphylococcal Count; LABC, Lactic Acid Bacteria count; TYMC, Total Yeast and Mould Counts.

**Table 2:** Microbiological counts (log CFU/g) of a different microbial group for minced meat of beaver (*n* = 6) *^a^*.

Microorganism *^b^*	Time of Storage			Weeks of Frozen
	24 h	7 Days	14 Days	11
TVC	4.80 ± 0.42 A	6.44 ± 0.65 B	8.08 ± 0.68 C	5.78 ± 0.17 B
PBC	3.62 ± 0.36 A	5.94 ± 0.61 B	8.33 ± 0.19 C	4.30 ± 0.39 D
EBC	3.34 ± 0.30 A	4.03 ± 0.37 B	5.70 ± 0.24 C	3.98 ± 0.32 B
EC	1.34 ± 0.26 A	1.52 ± 0.18 AB	1.70 ± 0.13 B	1.51 ± 0.23 AB
LABC	3.47 ± 0.49 A	4.69 ± 0.47 B	5.66 ± 0.14 C	4.18 ± 0.49 B
TSC	3.00 ± 0.25 A	3.70 ± 0.33 B	4.27 ± 0.50 C	3.74 ± 0.15 BC
TYMC	3.22 ± 0.19 A	4.02 ± 0.34 B	4.80 ± 0.09 C	3.70 ± 0.16 B

*^a^* Within rows, means with different letters are significantly different (*p* < 0.05). *^b^* TVC, Total Viable Count; PBC, Psychrotrophic Bacteria Count; EBC, *Enterobacteriaceae* Count; EC, *E. coli* count; TSC, Total Staphylococcal Count; LABC, Lactic Acid Bacteria count; TYMC, Total Yeast and Mould Counts.

## Data Availability

The data used to support the findings of this study are available from the corresponding author upon request.
